# A strategy for determining arterial blood gases on the summit of Mt. Everest

**DOI:** 10.1186/1472-6793-6-3

**Published:** 2006-03-08

**Authors:** Thomas F Catron, Frank L Powell, John B West

**Affiliations:** 1 Department of Medicine, University of California San Diego, La Jolla CA 92093-0623, USA

## Abstract

**Background:**

Climbers on the summit of Mt. Everest are exposed to extreme hypoxia, and the physiological implications are of great interest. Inferences have been made from alveolar gas samples collected on the summit, but arterial blood samples would give critical information. We propose a plan to insert an arterial catheter at an altitude of 8000 m, take blood samples above this using an automatic sampler, store the samples in glass syringes in an ice-water slurry, and analyze them lower on the mountain 4 to 6 hours later.

**Results:**

A preliminary design of the automatic sampler was successfully tested at the White Mountain Research Station (altitude 3800 m – 4300 m). To determine how much the blood gases changed over a long period, rabbit blood was tonometered to give a gas composition close to that expected on the summit (PO_2 _4.0 kPa (30 mmHg), PCO_2 _1.3 kPa (10 mmHg), pH 7.7) and the blood gases were measured every 2 hours for 8 hours both at sea level and 3800 m. The mean changes were PO_2 _+0.3 to +0.4 kPa (+2 to +3 mmHg), PCO_2 _0 to +0.13 kPa (+1 mmHg), pH -0.02 to -0.04, base excess -0.7 to -1.2 mM. In practice the delay before analysis should not exceed 4 to 6 hours. The small paradoxical rise in PO_2 _is presumably caused mainly by contamination of the blood with air.

**Conclusion:**

We conclude that automatic arterial blood sampling at high altitude is technically feasible and that the changes in the blood gases over a period of several hours are acceptably small.

## Background

There is good reason to believe that climbers at extreme altitudes, for example on the summit of Mt. Everest, are very close to their limit of tolerance to hypoxia. The combination of extreme hypoxemia combined with an extraordinary degree of respiratory alkalosis indicates a degree of physiological derangement of otherwise normal healthy people that is possibly unique.

During the 1981 American Medical Research Expedition to Everest (AMREE), alveolar gas samples taken on the summit, and at altitudes down to 8000 m, showed that the summit alveolar PO_2 _and PCO_2 _values were about 4.7 kPa (35 mmHg) and 1 kPa (7.5 mmHg), respectively. When these data were combined with measurements of base excess (BE) in venous blood drawn the following morning at an altitude of 8050, the calculated arterial blood gases on the summit were PO_2 _3.7 kPa (28 mmHg), PCO_2 _1 kPa (7.5 mmHg) and pH 7.7–7.8 [1]. No relevant measurements have been made on the summit in the last 25 years but some measurements of alveolar PO_2 _at 8000 m altitude are consistent with the AMREE results [2]. However, in the long-term low pressure chamber experiments Operation Everest II [3] and COMEX '97 [4], the results for the "summit" showed a lower alveolar PO_2_, higher arterial PCO_2_, and lower pH which can probably be explained by a lesser degree of acclimatization.

Apart from the physiological interest of understanding this extreme environment, the results are important for assessing the risks incurred by the many people who now attempt to climb Mt. Everest. Furthermore, severe hypoxemia is a feature of many patients in the intensive care unit and results may throw light on related problems here.

In addition to the blood acquired during rest, it is planned to collect samples immediately after exercise. These will indicate whether the arterial PO_2 _falls even further as might be expected from diffusion limitation across the pulmonary blood-gas barrier. Another important measurement will be blood lactate levels during rest and immediately after exercise. Previous measurements at extreme altitudes by Cerretelli and others [5] suggest that at altitudes over about 7500 m, there will be no increase in blood lactate concentrations as a result of exercise, a phenomenon known as the "lactate paradox" [6], see [7] for a recent review. This has never been tested under field conditions at these extreme altitudes. Finally, it will be of great interest to see to what extent the derangement of blood gases alters the chemistry profile of the blood. For example, does the severe respiratory alkalosis reduce the concentration of ionized calcium? Such a change may lead to tetany in the climber as possibly experienced by Habeler during the first ascent of Everest without supplementary oxygen. He stated "this feeling of being outside myself was interrupted for only a few moments. Cramp in my right hand bent my fingers together and tore me violently back to reality" [8].

At this time other groups are interested in obtaining arterial samples very high on Mt. Everest [9]. However, there are a number of limitations to drawing and analyzing blood in these extreme conditions. Arteropuncture requires some skill, and has more serious associated complications than venopuncture. It is probably not feasible to perform serial radial artery punctures at extreme altitude via conventional techniques. Furthermore, though portable blood gas and chemistry analyzers exist, they require temperature stability to function reliably. Such controlled conditions would not be immediately available to climbers at the time of sample acquisition. Therefore, the samples must be stored in some manner. It is known that arterial samples stored on ice (near 0°C) have slow internal gas and chemistry changes. However, this has never been formally validated for the periods of time required on Mt. Everest (up to 6 hours). Siggaard-Andersen [10] studied the changes in blood chilled in ice water but limited the studies to 3 hours. Kelman and Nunn [11] also looked at the time courses of blood gases but only in blood that cooled spontaneously, and only for up to one hour. Our approach to these problems is to insert an arterial catheter at an altitude of about 8000 m, take several blood samples above this including on the summit using an automatic sampler (actuated by the subject pressing a switch), store the samples in oiled glass syringes in an ice-water slurry, and analyze the samples lower on the mountain.

This study describes a proof-of-concept device for automated blood sampling which may be actuated by a climber without technical expertise. In addition we measured the changes in blood gases in blood with a gas composition near that expected on Everest that was stored near 0°C for up to 8 hours.

## Methods

### Automatic blood sampler

**Figure 1:**
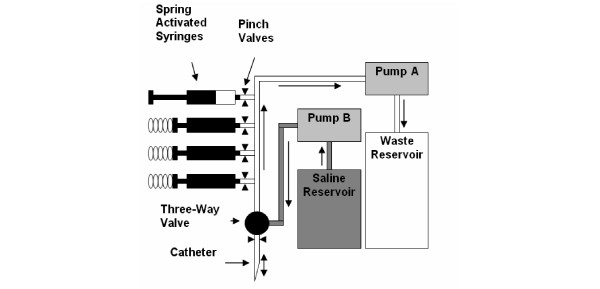
**Automatic arterial blood sampler**. Schematic representation of the automatic arterial blood sampler. See text for explanation.

A prototype automated arterial blood sampler (Figure 1) was designed and built in collaboration with the Department of Mechanical Engineering, University of California San Diego. This prototype was tested using animal blood in the laboratory and also in the field at the White Mountain Research Station (altitude 3800 m – 4300 m). In order to simulate the effects of normal arterial blood pressure, blood was exposed to a pressure of 17.3 kPa (130 mmHg) for some of these tests using a standard oversized sphygmomanometer. The device was evaluated for successful serial sample collection in the field and successful automated flushing of the system with heparinized saline (in order to prevent clotting of the tubing or catheter). The proof-of-concept device was built to show that automated blood sampling in the field is feasible. The construction of the final equipment that could be used in the hostile environment of the Everest summit would need much more development.

The sampling device (see Figure 1) relies on two small peristaltic pumps. First the system is primed with heparinized saline. When the climber presses a switch, the three-way valve is turned to connect the catheter to the syringes and pump A pulls blood from the radial artery towards the waste reservoir. After the saline in the dead space of the tubing has been cleared, a servo then opens a pinch valve at the syringe tip (located between the artery and the waste reservoir), and the spring loaded syringe is released, drawing 2 ml of blood. The pinch valve is then closed, and pumps B and A flush the main line with heparinized saline into the waste reservoir. Between sample collections, pump B flushes the catheter with heparinized saline at predetermined intervals to prevent coagulation. In the version shown in Figure 1 there are four syringes but this number can be increased as required. The sequence of operations is controlled by an electronic chip.

The apparatus consists of two separate units. First, a small box about 3 cm × 3 cm × 5 cm is located on the dorsal aspect of the climber's forearm a few cm above the wrist. This contains the three-way valve shown on Figure 1 and is connected to the indwelling radial arterial catheter. A tube then runs up the arm through the sleeve to a second box about 10 cm × 15 cm × 5 cm which is located on the front of the chest under the down clothing of the climber. The tube connecting the two boxes contains two polyethylene tubes and electrical wires to actuate the solenoid in the wrist box. The chest box is thermally insulated and contains the syringes shown in Figure 1 which are surround by an ice-water slurry. The box also contains the two peristaltic pumps and the two plastic bag reservoirs. In addition the box houses the solenoid-driven pinch valves, the activators to release the spring-loaded syringes, the chip controller, and the battery pack to power the units.

The total weight of the prototype was 2.2 kg. We used 5 ml syringes even though the sample was only 2 ml because this meant a shorter plunger movement for the springs. The polythene tubing had a 2.4 mm internal diameter, and the battery pack consisted of 4 rechargeable NiCad cells. However it should be emphasized that this was a proof-of-concept device and that producing a robust piece of equipment that would work in the more challenging environment of Mt. Everest would require additional design work. Extensive testing in a cold room would be necessary, and also with the subject exercising.

The proposed experimental plan is to have the radial artery catheter inserted by an M.D. in a well-lighted, warmed, special tent on the South Col (altitude about 8000 m). Prior to the expedition, the ulnar artery supply to the hand should be checked using an Allen test, and if there is any doubt about this angiography might be considered. The catheter and all tubing and other components of the equipment that would come into contact with the heparinized saline used for flushing the catheter would be carried up in presterilized packs and incorporated into the apparatus in the high tent. The operator who inserts the catheter would have an abundant supply of breathing oxygen. Arterial blood samples would be taken at this time during rest and exercise. Then the climber going to the summit would take two or three samples on the summit, and two or three more on the way down to the Col. When he/she returns to the South Col camp the catheter would be removed by the M.D., the blood samples placed in a special insulated box filled with an ice-water slurry, and the samples taken rapidly down to a lower camp where the analyzing equipment is located. A possible site for this would be at an altitude of about 6300 m in the Western Cwm where the main AMREE laboratory was located. Experience on AMREE showed that it was possible to get blood samples down from the South Col to this laboratory within about 4 hours. A possible blood analyzer is the i-Stat (Abbott, East Windsor, NJ). Special precautions need to be taken to prevent the reagents from freezing while in the laboratory or en route to it.

It is acknowledged that arterial catheter insertion in such a remote, hostile environment has risks. Close attention to sterile precautions needs to be given and the operator would have practiced the technique extensively. The subject would climb with a companion who would be aware of the possible complications. The wrist on the catheterized side might be lightly splinted, and a special glove, possibly with electrical heating, could be worn. At any time the catheter could be removed and bleeding stopped using local pressure.

### Blood studies

Rabbit blood was tonometered to a gas composition close to that expected on the summit of Mt Everest. The mean blood gas values after tonometering were: PO_2 _3.76 kPa (28.2 mmHg), PCO_2 _1.45 kPa (10.9 mmHg), pH 7.73. Rabbit blood was chosen because of the abundant supply of fresh blood. The blood gas changes due to metabolism in the storage syringes are caused by white blood cells and platelets, and rabbit blood has a similar concentration of these as human blood [12]. Samples were stored in lubricated glass syringes with matching plungers and barrels (as used in the prototype sampling device), and maintained at near 0°C in an ice-water slurry for 8 hours. An Instrumentation Laboratory Synthesis Clinical Analyzer and CO-Oximeter (Instrumentation Laboratory Company. Lexington, MA) were used to measure the PO_2_, PCO_2_, pH, base excess, and oxygen saturation. Measurements of oxygen saturation were only made at sea level because of technical problems.

Two different measurement protocols were used:

1. Measurements were made at time zero and subsequently every 2 hours to develop a time course of gas and chemistry changes.

2. Measurements were made at time zero and after 8 hours to determine the total gas and chemistry changes.

Protocol 1 was conducted at sea level (n = 8). Protocol 2 was conducted both at sea level (n = 7) and an altitude of 3800 m (n = 11) in order to determine whether ambient barometric pressures and therefore oxygen partial pressures would affect the changes observed in the stored blood.

### Statistics

Data from protocol 1 were analyzed using a one-tailed z-test to determine whether results at each time point varied significantly from zero change. Data from protocol 2 were analyzed in the same manner. Additionally, a two-tailed t-test was performed to determine significant differences between the samples analyzed at sea level and those measured at 3800 m. Significance was accepted at p < 0.05.

## Results

The prototype automatic sampling device successfully drew both non-pressurized and pressurized blood in the laboratory and in the field for up to 4 consecutive cycles (the number of syringes in this proof-of-concept model). The device also successfully performed periodic flushing of the attached catheter, and disposal of waste fluids into the waste reservoir.

**Figure 2:**
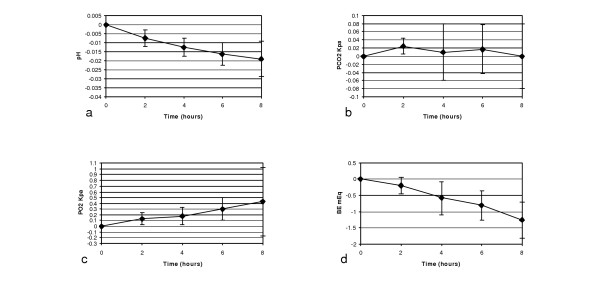
**Changes in arterial blood gases over 8 hours for protocol 1**. Mean changes over 8 hours, measured every 2 hours (protocol 1): (a) pH, (b) PCO_2_, (c) PO_2_, (d) Base Excess (BE). Note the different scales. PO_2_, pH, and BE show changes outside the error range for the blood analyzer. However, PCO_2 _variations are within the range of instrument error. Ranges represent ± 1 SD.

Time course studies (protocol 1) showed small but measurable and approximately linear changes for three variables: PO_2_, pH, and BE (Figure 2). These variables also showed statistically significant deviations from zero at all time-points (p values ranged from < 0.0001 to 0.022). It can be seen that there was a small steady rise in PO_2 _and small falls in pH and BE. During the time course studies, PCO_2 _changes were within the instrument error for blood gas electrodes and were not statistically significant (p = 0.23 to 0.52). O_2 _saturation data were incomplete due to problems with data collection during the experiment.

**Figure 3:**
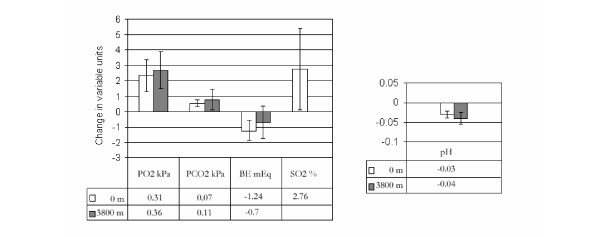
**Changes in blood gases over 8 hours for protocol 2**. Average blood gas and chemistry change over the course of 8 hours (protocol 2), and comparison of changes measured at sea level and at 3800 m. Units are dependent on the variable being measured. Note that SO_2 _data were collected only at sea level. Ranges represent ± 1 SD.

Measurements made at the beginning and end of the 8 hour period (protocol 2) showed measurable and statistically significant changes in all variables (p < 0.0001 to 0.01). However, the observed changes were small compared to the expected instrument error of blood gas analyzers. There were no significant differences between data collected at sea-level and data collected at 3800 m (p = 0.24 – 0.55) (Figure 3).

## Discussion

The prototype sampling device used in this study has several limitations, including relatively large size and weight and delicate components. However, as a proof-of-concept device it performed admirably. We were able to draw blood samples over the course of several hours while the device successfully flushed the lines at predetermined intervals. With further development, it will be possible to significantly refine the device, making it both smaller and more robust for field studies.

The tonometry data presented above indicate that there are small but measurable changes in blood stored at near 0°C for 8 hours. However, PCO_2 _and pH showed changes that are small compared to the accuracy of blood gas analyzers. For example, PCO_2_, which might be expected to rise due to continued metabolism, was seen to rise less than 0.11 kPa (0.8 mmHg) in all trials – a negligible change.

Changes in base excess are consistent with continued low level production of metabolic acids. As described below, it seems likely that these metabolic byproducts are responsible for some of the other observed changes in the samples.

Interestingly, rather than a decrease in PO_2 _and oxygen saturation, as may have been expected from continuing metabolism, both values were seen to rise slightly in the samples over time. These changes may be due to at least two factors. We used oiled glass syringes with matched plungers and barrels, which we feel to be the storage method most resistant to contamination by ambient air. However, in an environment with a relatively high ambient PO_2 _there may be no way to completely eliminate contamination over several hours. In a low pressure environment (such as on Mt. Everest), this contamination might be expected to be less due to the decreased PO_2 _in the air. It is interesting to note though, that there was no significant difference in the changes in PO_2 _at 3800 m compared to sea level.

Additionally, as small amounts of metabolic acid are produced through continuing metabolism of leucocytes and platelets, a rise in PO_2 _of up to 0.13 kPa (1 mmHg) can be attributed to the effects of this acid on hemoglobin-oxygen dissociation [13,14]. Hence, as pH drops and BE becomes increasingly negative in these samples, the observed PO_2 _increases slightly because of the reduction of the P_50_. In practice, the delay between sample acquisition and analysis should be less than 6 hours. The general conclusion is that the overall changes in these samples were small enough to allow relatively accurate and reliable assessment of blood gas composition over the anticipated period of time.

## Conclusion

Arterial blood gases on the summit of Mt. Everest would give exceptionally interesting information about this extreme environment. The automated arterial blood sampler that has been described here is technically feasible although of course the whole experiment is very ambitious. The changes in the blood gases over a period of several hours are acceptably small. The device might also be useful in other extreme environments.

## Authors' contributions

TC carried out the field studies and the blood measurements. FP participated in the design of the study and helped with the field studies. JW conceived of the study, and participated in its design and coordination and help to draft the manuscript. All authors read and approved the final manuscript.
